# Residual Cognitive Deficit in Adults with Depression who Recovered after 6-month Treatment: Stable versus State-Dependent Markers

**DOI:** 10.4021/jocmr2009.10.1266

**Published:** 2009-10-16

**Authors:** Charles Lung-Cheng Huang

**Affiliations:** a Department of Psychiatry, National Taiwan University Hospital Yun-Lin Branch, Taiwan; b Institute of Medicine, Kaohsiung Medical University, Taiwan

## Abstract

**Background:**

Knowledge of depression-related disturbances in cognitive functioning is advancing, but little is known about the cognitive response to treatment for major depression, especially in younger adults. This study investigated the deficits in multiple cognitive domains in middle-aged patients with major depressive disorder (MDD), using a prospective follow-up study design.

**Methods:**

The sample consisted of 13 medication-free MDD patients and 13 education- and age-matched healthy controls. All subjects were administered clinical measures as well as a comprehensive neurocognitive test battery aimed at assessing multiple cognitive domains at the time of recruitment. Patients remitted after 6 months following treatment repeated the neurocognitive assessment.

**Results:**

There were significant differences between the depressed subjects and controls at baseline. MDD patients with remitted symptoms still showed significant deficits in executive function and motor function, but not in memory or attention domains. Patients had significant improvement in memory and attention domains only, once their depressive symptoms had subsided; while executive functioning as well as motor functioning remained unchanged.

**Conclusions:**

Executive functioning and motor functioning deficits might be stable vulnerability indicators for MDD, and memory and attention impairment might serve as state-dependent indicators for MDD.

**Keywords:**

Major depressive disorder; Remission; Residual; Cognitive deficits; Follow-up

## Introduction

Depression-related disturbances in cognitive function have been demonstrated in a range of domains [1,2]. However, it is still unclear if the cognitive impairment is a state characteristic of major depressive disorder (MDD), or whether the cognitive deficit persists upon recovery from depressive episodes. The findings across previous studies have varied somewhat, but suggest that a substantial proportion of patients with a history of depression exhibit persistent cognitive impairment, particularly in memory [3], attention [4], and executive functioning [5]. The use of testing before and after recovery is a potentially more powerful method of identifying and distinguishing state- from trait-related cognitive deficits [6]. Using this design, a small number of studies have reported improvement in cognitive functioning, such as memory [7-11], attention [10], executive function [9,11], and psychomotor function [9], after treatment. In contrast, some authors found that residual cognitive deficits involving memory [12-14], executive function [10,13], attention [13,15], and psychomotor function [10,12], persisted upon clinical recovery.

The controversial results may due to some methodological limitations in these longitudinal studies. For example, using inadequate definitions of recovery [7,8,15], failing to show that task performance was within the normal range at recovery [9,11], not controlling for the potential effects of age and educational level [9,11], and not controlling for the potential effects of medication and electroconvulsive therapy [9,10,14,15]. In addition, the majority of these studies focused on elderly individuals [10-13]. Although knowledge of residual cognitive dysfunction in elderly MDD patients is increasing, significant gaps remain in our understanding of the cognitive response to treatment in younger adult depression.

The present study investigated the deficits in multiple cognitive domains of adults with depression with a prospective follow-up, case-control design. The main objective was to examine whether cognitive deficits in MDD patients improve with successful treatment of depression, as well as to determine stable versus state-dependent markers of MDD.

## Materials and Methods

### Subjects and design

This was a prospective follow-up, case-control study. Thirteen patients (aged 18-50 years) with a diagnosis of non-psychotic, unipolar MDD from the DSM-IV, were recruited. In addition, they had scored at least 16 on the 17-item Hamilton Depression Rating Scale (HAM-D-17) [16] and were entirely psychotropic medication-free currently and for at least six weeks before recruitment. Subjects were excluded if they had a history of any neurological or major medical illness, or significant alcohol or substance abuse. Thirteen education- and age-matched healthy controls were recruited from the community by advertisement. These volunteers were interviewed by a senior psychiatrist using the Chinese version of the Mini International Neuropsychiatric Interview (MINI) [17] to exclude individuals with mental illnesses.

Severity of depression was rated using the 17-item Hamilton Rating Scale for Depression (HAM-D-17) for all subjects. A comprehensive neurocognitive test battery, including the Wechsler Memory Scale-Revised (WMS-R) [18], Wisconsin Card Sorting Test (WCST) [19], Continuous Performance Test (CPT) [20], and Finger Tapping Test (FTT) [21], was used to assess memory, attention, executive functioning, and motor functioning domains, respectively. The battery was administered at the time of recruitment as a baseline measure (assessment I). The same battery was used with the group of patients whose depressive symptoms had remitted (scoring less than 7 on the HAM-D) after six months of treatment (assessment II). During the follow-up period, patients received a standard treatment protocol with SSRI only. All subjects signed an informed consent statement. The Ethical Committee for Human Research of local hospital had approved the study protocol.

### Data analyses

Rates and proportion with a two-tailed 95% CI were calculated for the variables of interest. Cross-table chi-square analysis was used to compare sex, education level, and marital status of the MDD patients and healthy controls. The significance of differences in cognitive performance between the two groups was calculated with the Mann-Whitney Test. The significance of differences in the cognitive measures of the MDD subjects between assessment I and assessment II was tested using a Wilcoxon signed ranks test. All data were analyzed using the SPSS for Windows package, Release 10.0.

## Results

The demographic profiles and HAM-D scores for the MDD patients and controls are shown in Table 1. The average age of the patients was 37.2 years. There was no significant difference in gender, education level, or age between the patients and control groups.

**Table 1:** Demographic data and HAM-D scores for MDD patients and controls

	MDD patients		Normal controls		‡Z	*p* value
	Mean ± SD	N	Mean ± SD	N		
**Sex**						0.69
Male		4		6		
Female		9		7		
**Education level**						0.22
Below junior high school		3		0		
Above junior high school		10		13		
**Age**	37.19 ± 12.62	13	38.92 ± 12.61	13	-0.51	0.61
†**HAM-D-17**	23.23 ± 6.10	13	0.38 ± 0.51	13	-4.41	< 0.01

†HAM-D-17:17-item Hamilton Rating Scale for Depression ‡Mann-Whitney Test was used to compare the ages and HAM-D scores in both groups Cross table chi-square was used to compare the sex and education level

The HAM-D scores and neurocognitive measures for the MDD patients and controls are shown in Table 2. Compared to the controls, the depressed patients were significantly impaired in several cognitive domains, including memory (as measured by the WMS-R), executive function (as measured by the WCST), motor function (as measured by the FTT), and attention domains (as measured by the CPT) at assessment I. MDD patients with remitted symptoms still showed significant deficits in executive function and motor function, but not in the memory or attention domains at assessment II.

**Table 2:** HAM-D scores and neurocognitive measures for MDD patients and controls

	MDD Assessment I		MDD Assessment II		Normal Controls		‡Assessment I		‡Assessment II		§ Assessment I	
							vs.		vs.		vs.	
							Normal Controls		Normal Controls		Assessment II	
	Mean ± SD	N	Mean ± SD	N	Mean ± SD	N	Z	*p* value	Z	Sig.	Z	*p* value
†**HAM-D-17**	23.23 ± 6.10	13	2.38 ± 2.84	13	0.38 ± 0.51	13	-4.41	< 0.01	-1.91	0.06	-3.18	< 0.01
†**WMS-R**												
Verbal MEM.	81.33 ± 16.90	13	90.38 ± 15.23	13	96.18 ± 9.26	13	-2.53	< 0.05	-1.54	0.13	-2.33	< 0.05
Visual MEM.	78.92 ± 22.63	13	106.13 ± 20.99	13	111.18 ± 16.56	13	-2.93	< 0.01	-0.74	0.46	-2.52	< 0.05
General MEM.	76.08 ± 22.27	13	93.75 ± 14.92	13	100.55 ± 11.03	13	-2.90	< 0.01	-1.49	0.14	-2.38	< 0.05
Attention/Concentration	91.75 ± 18.22	13	99.70 ± 16.79	13	104.00 ± 14.13	13	-2.34	< 0.05	-1.14	0.25	-2.02	< 0.05
Delayed Recall	82.42 ± 22.67	13	102.25 ± 17.69	13	104.00 ± 15.40	13	-2.40	< 0.05	-0.37	0.71	-2.38	< 0.05
†**FTT**												
Dominant hand	30.69 ± 15.03	13	31.99 ± 16.46	13	59.24 ± 10.85	13	-3.59	< 0.01	-3.3.2	<0.01	-1.61	0.11
†**WCST**												
Perseverative Errors	10.50 ± 4.36	13	9.42 ± 3.40	13	4.50 ± 7.12	13	-2.37	< 0.05	-2.10	<0.05	-1.53	0.13
†**CPT**												
d’ Masked	3.01 ± 0.94	13	3.47 ± 0.93	13	3.88 ± 0.64	13	-2.32	< 0.05	-1.00	0.32	-2.51	< 0.05

†HAM-D-17:17-item Hamilton Rating Scale for Depression; WMS-R: Wechsler Memory ScaleRevised; CPT: Continuous Performance Test; FTT: Finger Tapping Test; WCST: Wisconsin Card-Sorting test ‡Mann-Whitney Test §Wilcoxon signed ranks test

## Discussion

Our study demonstrated that remitted depressed patients with baseline cognitive impairment had experienced improvement in specific cognitive domains, i.e., memory as well as attention, and could reach the same performance levels as the controls. This is consistent with a number of recent studies of major depression [7,8]. This result echoed the findings of studies of elderly patients by Butters et al [11] and Beats et al [10]. In contrast, Neu et al assessed middle-aged depressed patients with a melancholic subtype and reported that after sustained remission [14], the patient group still performed significantly worse in verbal memory and verbal fluency compared to the controls. Other studies found persistent memory or attention dysfunction more often, and found these phenomena in older patients, mostly with endogenous depression [2].

The other finding in our study was that cognitive impairment in the executive functioning and motor functioning domains persisted even after the depressed symptoms had recovered. This result adds to the body of evidence that MDD may yield some residual cognitive deficits even with successful treatment [5,15]. Beats et al investigated elderly depressed patients on tests sensitive to frontal lobe dysfunction [10], and found that measures of simple and choice reaction times, perseveration on the set-shifting task, and verbal fluency did not fully recover. Nebes et al also found that decreased working memory and processing speed mediated cognitive impairment in elderly patients [12]. The processing resource decrement persisted after remission of the depression, and thus may be a trait marker of geriatric depression. Some authors suggest that working memory deficits in depression are due to persistent deficits in selective attention (state-independence) have been related to persistent abnormalities in the prefrontal cortex [4,15]. Furthermore, the findings across studies support the hypothesis that major depression is frequently associated with cognitive dysfunction and that the underlying defect is in the subcortical/frontal lobe neural circuitry, i.e., the frontostriatal dysfunction [10,22,23]. However, studies that did find an association between lesions and cognitive function more often found this phenomenon in depressives, mostly with a late-onset of the illness [23-26]. Although microvascular pathology may account for the persistent cognitive impairment seen in elderly patients with depression [27], the contribution of such a process to the residual cognitive impairment in younger depressives remains uncertain.

In sum, our study implies that there might be a specific profile of cognitive deficits as stable vulnerability indicators for MDD, and some other cognitive impairment may serve as state-dependent indicators for MDD. A generalization of our results must be viewed with caution, considering the relatively small sample size. Clearly, further researches need to be undertaken with a larger sample size and more parameters to clarify the specific cognitive deficit of remitted MDD patients.
